# Association between serum tricosanoic acid and cognitive function in older adults: findings from the NHANES and GEO databases

**DOI:** 10.3389/fnagi.2025.1534303

**Published:** 2025-03-20

**Authors:** Ti Yang, Yue Zhang, Zeen Cai, Ying Wang, Shengqiong Deng

**Affiliations:** ^1^Department of Clinical Pharmacy, Gongli Hospital of Shanghai Pudong New Area, Shanghai, China; ^2^Department of Clinical Pharmacy, Jimo People’s Hospital, Qingdao, Shandong, China; ^3^School of Gongli Hospital Medical Technology, University of Shanghai for Science and Technology, Shanghai, China; ^4^Shanghai Health Commission Key Lab of Artificial Intelligence (AI)-Based Management of Inflammation and Chronic Diseases, Department of Clinical Laboratory, Gongli Hospital of Shanghai Pudong New Area, Shanghai, China

**Keywords:** Alzheimer’s disease (AD), tricosanoic acid, GEO, NHANES, older adults

## Abstract

**Introduction:**

With global aging, dementia prevalence rises. While long-chain saturated fatty acids show anti-cognitive decline potential, serum tricosanoic acid (C23:0)’s role in brain regions and cognition remains unclear.

**Methods:**

To confirm the association between C23:0 and cognition in the population, we analyzed gene expression data from the Alzheimer’s disease (AD) brain gene chip data set (GSE118553) available in the Gene Expression Omnibus (GEO) database. Additionally, we examined data from 1,127 adults aged 60 years and older who participated in the National Health and Nutrition Examination Survey (NHANES) between 2011 and 2014. To explore potential metabolic pathways and mechanisms linking C23:0 to cognitive aging, the computational platform METAFlux was employed.

**Results:**

Differential gene expression analysis identified 335 downregulated and 477 upregulated genes in AD frontal cortex. Metabolite analysis showed 20 upregulated and 37 downregulated nutrients (including C23:0) in AD vs. controls. Population-level analysis (NHANES, *n* = 1,127) confirmed higher serum C23:0 associated with better cognitive function.

**Discussion:**

This study provides strong evidence for frontal cortex-specific reduced C23:0 in AD and highlights its potential as a serum cognitive marker.

## 1 Introduction

The economic burden of cognitive decline and dementia is substantial, making it important to find new strategies to prevent cognitive impairment, particularly as the global population continues to age (GBD 2019 Dementia Forecasting Collaborators, 2022). Fatty acids, derived from both dietary intake and metabolic processes, play a significant role in cognitive function (Baierle et al., 2014). Long-chain polyunsaturated fatty acids have been widely reported to exert neuroprotective effects, potentially slowing cognitive decline and reducing the risk of dementia (Andriambelo et al., 2023; Dhillon et al., 2023; Oulhaj et al., 2016; Qiang et al., 2024; Snowden et al., 2017; Tang et al., 2020; Wei et al., 2023).

However, the precise effects of long-chain saturated fatty acids (SFAs) on cognitive function remain inconclusive. While adherence to a Mediterranean diet, characterized by a high intake of monounsaturated fatty acids from olive oil (Berr et al., 2009; Solfrizzi et al., 2006) and reducing intake of foods high in saturated fatty acids (SFAs) (Kalmijn et al., 2004; Morris et al., 2004) may be associated with a decreased risk of developing dementia (Ballarini et al., 2021; Ogbodo et al., 2022). However, some studies have reported that individuals with high levels of SFA are less likely to develop AD (Kotlega et al., 2021; Ronnemaa et al., 2012). Recently, plasma long-chain SFAs containing 20 or more carbon atoms have attracted increasing attention for their potential health benefits (Forouhi et al., 2014; Lemaitre and King, 2022; Li et al., 2020; Ronnemaa et al., 2012; Shen et al., 2024).

Tricosanoic acid (C23:0) is a long-chain SFA composed of 23 carbon atoms. However, its impact on cognition remains uncertain, with conflicting findings regarding the effects of serum long-chain SFAs on cognitive function (Dhillon et al., 2023; Kotlega et al., 2021; Shen et al., 2024). Additionally, the relationship between these fatty acids and their expression in specific brain regions has yet to be fully elucidated. In this study, we aimed to investigate the differences of C23:0 expression in frontal cortex between healthy individuals and those with cognitive impairment. To achieve this, we analyzed gene expression data from the Alzheimer’s disease (AD) brain gene chip dataset (GSE118553) available in the Gene Expression Omnibus (GEO) database. This dataset provides a comprehensive view of gene expression changes in the frontal cortex, a brain region important for cognitive and memory function.

**FIGURE 1:**
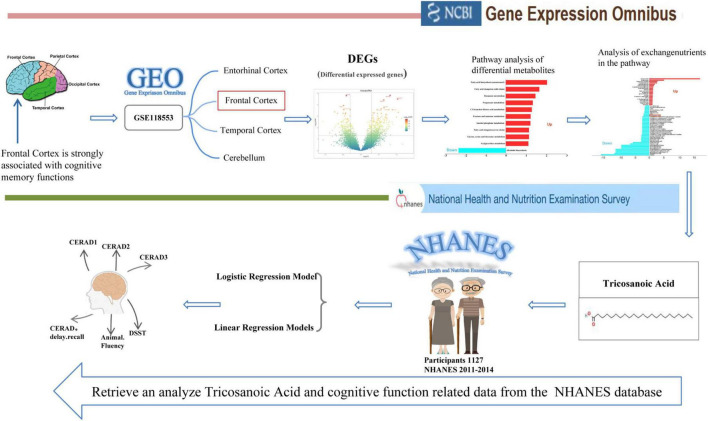
Flow chat for the study design. Gene Expression Analysis indicates differential expression of C23:0 in the frontal cortex between healthy individuals and those with cognitive impairment using data from the AD brain gene chip dataset (GSE118553) from the GEO database. Correlation Analysis illustrates the relationship between serum C23:0 levels and cognitive performance in adults aged 60 years and older based on data from the NHANES program (2011–2014).

Furthermore, we used data from the NHANES program to further investigate the connection between serum C23:0 levels and cognition at the population level. NHANES is a nationally representative program designed to assess the health and nutritional status of non-institutionalized U.S. civilians through household interviews, physical exams, and laboratory assessments based on a complex probability sampling design. Specifically, our analysis focused on adults aged 60 years and older, using data from the NHANES dataset (2011–2014). By integrating these two datasets, we aimed to provide a more comprehensive understanding of the relationship between serum C23:0 levels and cognitive function in older adults (Figure 1).

## 2 Materials and methods

### 2.1 General research design

This study was conducted in two parts. First, we analyzed AD brain gene chip expression data from the GEO database to identify significantly altered metabolites. Second, we explored the association between serum C23:0 levels and cognitive function using data from the NHANES.

### 2.2 Metabolite screening

#### 2.2.1 Data acquisition

Genomic data from the AD brain gene chip dataset GSE118553, was obtained from the GEO database (Patel et al., 2019). This dataset is based on the Illumina HumanHT-12 V4.0 expression beadchip platform (GPL10558), includes microarray data from brain regions affected by AD pathology (entorhinal cortex, temporal cortex, and frontal cortex) as well as an unaffected region (cerebellum). Given the frontal cortex’s role in cognitive and memory functions (Badre and Wagner, 2007; Chang et al., 2022; Fletcher and Henson, 2001; Miller, 2000; Zamzow et al., 2019), our study focused specially on this reagion. In total, there were 23 cognitively unimpaired controls and 73 AD-diagnosed individuals included in the differential analysis. As the data were obtained from a public database, ethical approval was not required.

#### 2.2.2 Differential gene expression analysis

The GSE118553 dataset from the frontal cortex was normalized using the “limma” package within R4.3.2 (Ritchie et al., 2015). Differential gene expression analysis was then conducted to compare the control group and the AD group, with significance thresholds set at |log_2_FC| > 0.1 and *P* < 0.05. The results were visualized using a volcano plot to highlight significantly upregulated and downregulated genes.

#### 2.2.3 Differential analysis of metabolic pathways and metabolites for DEGs

METAFlux is a computational platform for flux balance analysis and metabolic modeling of microbial and eukaryotic organisms.^1^ The “METAFlux” package in R4.3.2 was used to obtain the Human1 model data (Robinson et al., 2020). Metabolic pathway activity analysis was conducted to identify significantly altered metabolic pathways in AD. Additionally, changes in 1,648 exchange metabolites between the control group and the AD group were examined. A *t*-test was then performed for each metabolite in both groups to identify significantly altered substances (Long and Reed, 2017).

### 2.3 Observational study

#### 2.3.1 Study design and eligibility criteria

NHANES, conducted by the Centers for Disease Control and Prevention (CDC), estimates the conditions and potential risk factors of health and nutrition among the U.S. non-institutionalized civilian population. This survey employs a complex probability sampling design, combined with extensive household interviews, physical examinations, and laboratory assessments. Our investigation was structured as a cross-sectional study, leveraging comprehensive data from the NHANES (2011–2014). Our analysis centered on a cohort of adults aged 60 and older. We specifically utilized data from two NHANES cycles (2011/2012, 2013/2014), which included both serum C23:0 measurements and cognitive function assessments measures. The NHANES protocol was meticulously reviewed and approved by the National Center for Health Statistics’ Ethics Review Board, underscoring adherence to stringent ethical standards. In strict compliance with these guidelines, informed consent was obtained in writing from all participants prior to their involvement in the survey. This study adhered to the Strengthening the Reporting of Observational Studies in Epidemiology–Nutritional Epidemiology (STROBE-nut) reporting guidelines (Lachat et al., 2016).

**FIGURE 2:**
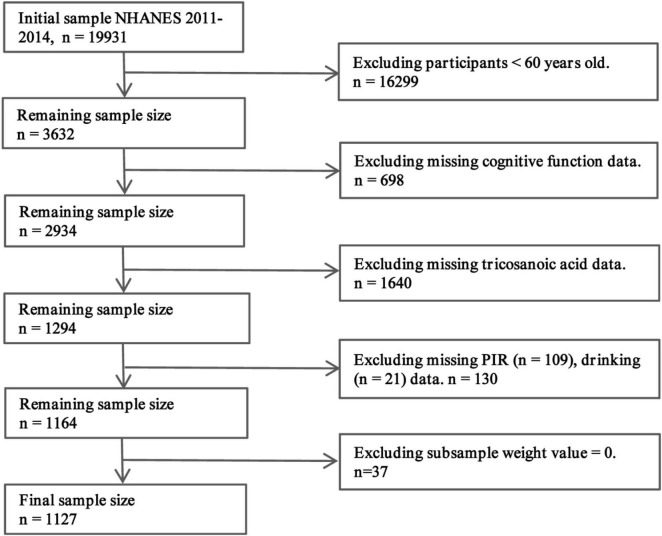
Path diagram of the patient selection process. From the initial pool of 19,931 participants in the 2011–2014 NHANES dataset, we excluded 16,299 individuals who were under 60 years old and 698 who did not have cognitive function data. Additionally, 1,640 were removed due to incomplete C23:0 laboratory data, 130 lacked PIR and drinking information, and 37 had a subsample weight of “0.” After this thorough selection process, a final analytical sample of 1,127 individuals was obtained, ensuring the integrity and reliability of our analyses.

Of the 19,931 participants in the 2011–2014 NHANES dataset, 16,299 individuals under 60 years of age and 698 participants without cognitive function data were excluded. Subsequently, 1,640 individuals with incomplete laboratory data on C23:0 levels and 130 participants with missing PIR and drinking data were removed. Lastly, 37 participants with a subsample weight value of “0” were also excluded. The final analytical sample consisted of 1,127 participants. Figure 2 shows the process of participant selection.

#### 2.3.2 Measurement of serum tricosanoic acid

All participants examined in the morning session at the mobile examination center were eligible for inclusion. Serum samples were obtained and stored at −30°C until analysis at the Division of Laboratory Sciences, National Center for Environmental Health. C23:0 levels were measured using electron capture negative-ion mass spectrometry detected. A comprehensive quality assurance program was implemented to ensure the integrity and reliability of the data, including blind split sample performance collected during “dry run” sessions. Details of laboratory methodology, quality assurance, and monitoring can be found at: https://wwwn.cdc.gov/Nchs/Nhanes/2011-2012/FAS_G.htm and https://wwwn.cdc.gov/Nchs/Nhanes/2013-2014/FAS_H.htm#LBXTSA (Fan et al., 2021; Janssen and Kiliaan, 2014).

#### 2.3.3 Cognitive assessment

Cognitive function evaluations were systematically administered to participants aged 60 and older in the NHANES by trained interviewers during the face-to-face interview at designated mobile examination center. The assessments included word learning and recall modules based on the CERAD, animal fluency test and DSST. The word learning and recall module of CERAD was used to ascertain the learner’s immediate and delayed learning ability of new words. In CERAD recall learning, participants read 10 ordered words aloud in three sessions, with the order changing in each session. The maximum score for both the word learning and memory modules was 10. The CERAD memory test was administered after the animal fluency test and DSST. The animal fluency test was designed to test the executive ability of the participants and required the participants to enumerate the names of animals within 1 min, with one point awarded per correct response. Processing speed, sustained attention and working memory were assessed using the DSST, a performance module from the Wechsler Adult Intelligence Scale. In all tests of cognitive function, higher scores indicate better cognitive function.

#### 2.3.4 Covariates

The analysis accounted for a comprehensive array of covariates to adjust for potential confounding factors. These covariates encompassed essential demographic characteristics such as sex, age categorized into three distinct groups: (1) 60–69 years, (2) 70–79 years, and (3) 80 years and older, as well as self-reported race/ethnicity defined as non-Hispanic white, non-Hispanic black, Mexican American, and other. Education level was considered with the following classifications: below high school, high school or General Educational Development (GED) diploma, and above high school education. Socioeconomic status was approximated using PIR. Lifestyle factors, including alcohol consumption, were also controlled for, with participants grouped into non-drinkers, those consuming 1–5 drinks per month, 5–10 drinks per month, and 10 or more drinks per month. Lastly, the presence of sleep disorders was included as a covariate to address potential effects on cognitive function.

### 2.4 Statistical analysis

All data were analyzed using R4.3.2. A comprehensive descriptive analysis was performed for all participants; continuous variables were presented as medians (25th and 75th percentiles), while categorical variables were presented as percentages (%). Categorical variables were evaluated using the chi-square test to determine any significant associations or differences between groups. For continuous variables, given the potential for non-normal distribution, the non-parametric Wilcoxon rank-sum test was employed to compare medians between two independent groups. A logarithmic transformation was applied to approximate a normal distribution of serum C23:0 levels, and the population was divided into four groups according to quartiles. The association between serum C23:0 levels and CERAD word learning and recall scores was analyzed using the weighted logistic regression model. The results were reported as the OR and its 95% confidence interval (95% CI) and *P*-value. Linear regression models were used to evaluate association with CERAD word learning total score, animal fluency score, and DSST score. The results were reported as beta coefficients and their standard error (SE) and *P*-value. To probe the connection between serum C23:0 levels and cognitive function, we sequentially applied three logistic regression models. Initially, Model 1 examined the unadjusted association, while Model 2 introduced age-adjustment to refine the analysis by accounting for this key demographic factor. Model 3 included adjustments for serum C23:0 levels, age, race, educational level, PIR, alcohol intake, and sleep disorders. Statistical significance was set at *P* < 0.05, a standard for evaluating data associations or differences’ reliability.

## 3 Results

### 3.1 Metabolite screening

#### 3.1.1 Screening for DEGs between the control group and the AD group

**FIGURE 3:**
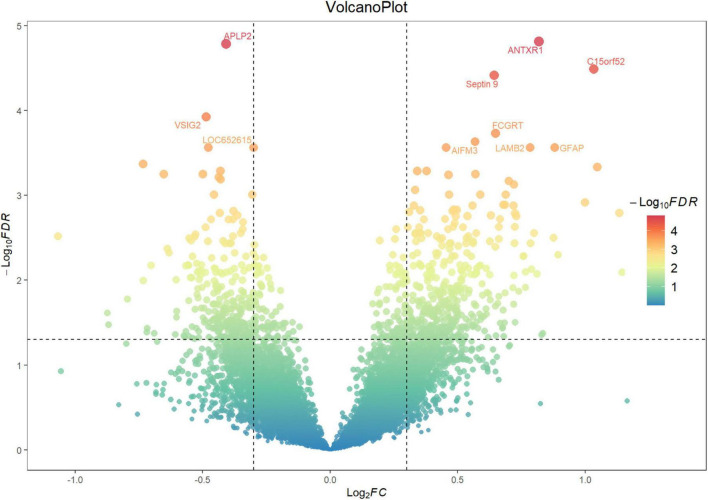
Volcano plot showing the number of differentially expressed genes. The x-axis represents the log2 FC in gene expression between the AD and control groups, while the y-axis represents the −log10 *p*-value. Each point represents a single gene. Genes with ||*log*_2_FC|≥0.3(*p* < 0.05) are highlighted, with those showing ||*log*_2_FC|≥2 further labeled in red with gene names. Significant upregulated genes are located in the upper right quadrant, and significant downregulated genes are located in the upper left quadrant.

A total of 812 genes were identified as differentially expressed between the control group (*n* = 23) and the AD group (*n* = 73), with 335 genes downregulated and 477 genes upregulated in the AD group compared to the control group, as shown in Figure 3 and Supplementary Table S1.

#### 3.1.2 Metabolic pathway and metabolite activity analysis of DEGs

**FIGURE 4:**
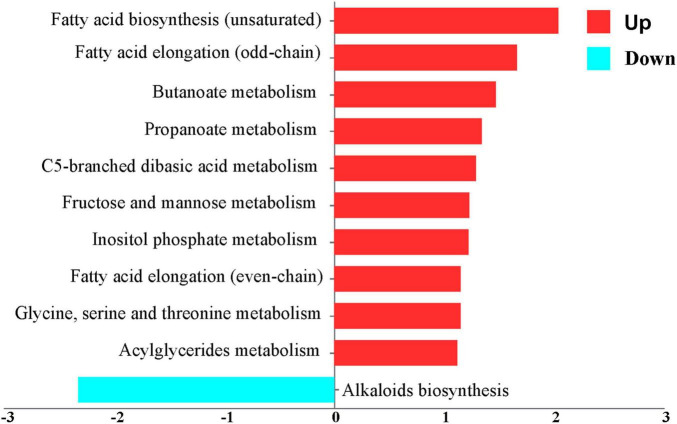
Metabolic pathway enrichment plot. A negative x-score indicates that the activity is decreased (blue). A positive x-score indicates that the activity is increased (red).

**FIGURE 5:**
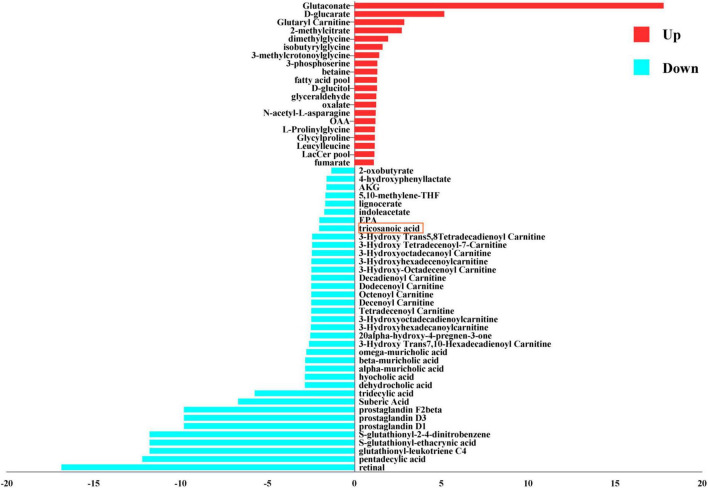
Metabolite activity enrichment plot. A negative x-score indicates that the activity is decreased (blue). A positive x-score indicates that the activity is increased (red).

Metabolic pathway activity analysis, employing the criteria of | changes | ≥ 1 and *P* < 0.05, indicated that the activity of DEGs increased in 10 pathways and decreased in 1 pathway, as depicted in Figure 4 and Supplementary Table S2. The upregulated pathways include fatty acid biosynthesis (unsaturated), fatty acid elongation (odd-chain), butanoate metabolism, propanoate metabolism, C5-branched dibasic acid metabolism, fructose and mannose metabolism, inositol phosphate metabolism, fatty acid elongation (even-chain), Glycine, serine and threonine metabolism, and acylglyceride metabolism. The downregulated pathway is alkaloid biosynthesis. A *t*-test was used to analyze the metabolites exchanged between the control and the AD groups. It indicated that 20 nutrients showed an increase, while 37 nutrients showed a decrease, based on the criteria | changes | ≥ 1 and *P* < 0.05. The increased nutrients include glutaryl carnitine, fatty acid pool, and fumarate. The decreased nutrients include tricosanoic acid, decadienoyl carnitine, octenoyl carnitine, tridecylic acid, and suberic acid, as illustrated in Figure 5 and Supplementary Table S3.

### 3.2 Observational study

#### 3.2.1 General characteristics of NHANES

Table 1 meticulously outlines the demographic and baseline characteristics of the study population. It is noteworthy that the mean age of participants at screening was 68 years, with the majority of participants (54%) being between the ages of 60 and 69 years. Among these participants, 580 (80%) were non-Hispanic white. Furthermore, 84% of the participants had obtained at least a high school education. The majority of participants reported consuming alcohol, with only 27% indicating that they were non-drinkers. Additionally, 13% of participants reported experiencing sleep disorders. Participants had a mean C23:0 level of 29 μmol/L. The data for this analysis were obtained from the NHANES dataset from 2011 to 2014. Regarding cognitive function, the median CERAD score for the three-word learning trials (maximum 30) was 20, while the median CERAD score for the delay recall test was 7. Additionally, the median animal fluency test and DSST scores were 18 and 53, respectively (Table 1).

**TABLE 1:** Background characteristics of older adults 60 years and above, NHANES 2011–2014.

Demographic data	*n* (unadjusted)	Percentage (adjusted)^a^
Total	1,127	100%
**Sex**		
Female	586	55%
Male	541	45%
**Age group**		
60–69 years	567	54%
70–79 years	316	26%
80 + years	244	20%
**Race**		
Other race	213	8.3%
Mexican American	103	3.4%
Non-Hispanic Black	231	8.4%
Non-Hispanic White	580	80%
**Educational_level**		
Below high school	273	16%
High school/GED	258	21%
Above high school	595	63%
PIR.group		
<5	915	74%
≥5	212	26%
**Alq group**		
Non-drinker	358	27%
1–5 drinks/month	544	49%
5–10 drinks/month	51	5.1%
10 + drinks/month	172	18%
**Sleep.disorder**		
No sleep disorder	980	87%
Sleep disorder	147	13%
	**Median**	**25th, 75th percentiles**
Tricosanoic acid (μmol/L)	29	25, 35
Age (years)	68	63, 74
PIR	3.14	1.72, 5.00
CERAD1	5.00	4.00, 6.00
CERAD2	7.00	6.00, 8.00
CERAD3	8.00	7.00, 9.00
CERAD.total	20.0	17.0, 23.0
CERAD.delay.recall	7.00	5.00, 8.00
Animal.fluency	18.0	14.0, 22.0
DSST	53	42, 64

^a^Adjusted for sampling weight, strata, and primary sampling unit.

#### 3.2.2 Association between serum tricosanoic acid levels and cognitive function

Serum C23:0 levels were positively correlated with cognitive function using the Wilcoxon rank-sum test for complex survey samples. A correlation was observed between the serum C23:0 levels of the participants and their performance on a range of cognitive tasks. Specifically, higher serum C23:0 level was associated with superior performance on the CERAD word learning trials, the CERAD delayed recall test and the DSST. However, no significant increase was observed in the score on the animal fluency test with increasing levels of serum C23:0. Additionally, no significant sex-based differences were observed in these associations (Table 2).

**TABLE 2:** Association between serum tricosanoic acid levels and cognitive function.

Characteristic	Q1	Q2	Q3	Q4	*p*-value^a^
**Tricosanoic acid (μmol/L)**					
Tricosanoic acid (μmol/L)	21 (19, 24)	27 (26, 28)	32 (31, 33)	40 (37, 44)	<0.001
Men only	21 (19, 24)	27 (26, 28)	32 (30, 33)	40 (37, 43)	<0.001
Women only	21 (19, 24)	27 (26, 28)	32 (31, 34)	40 (37, 44)	<0.001
CERAD1	5.00 (4.00, 6.00)	5.00 (4.00, 6.00)	5.00 (4.00, 6.00)	5.00 (4.00, 6.00)	0.012
CERAD2	7.00 (6.00, 8.00)	7.00 (6.00, 8.00)	7.00 (6.00, 8.00)	8.00 (7.00, 9.00)	<0.001
CERAD3	8.00 (6.00, 9.00)	8.00 (7.00, 9.00)	8.00 (7.00, 9.00)	8.00 (7.00, 9.00)	<0.001
CERAD.total	19.2 (16.7, 22.0)	20.0 (18.0, 23.0)	20.0 (17.0, 23.0)	21.0 (19.0, 24.0)	<0.001
CERAD.delay.recall	6.00 (4.00, 8.00)	6.00 (5.00, 8.00)	7.00 (5.00, 8.00)	7.00 (5.00, 9.00)	<0.001
Animal fluency: score total	17.0 (14.0, 21.0)	17.0 (13.0, 20.0)	19.0 (15.0, 22.0)	18.0 (15.0, 23.0)	0.077
DSST	48 (37, 59)	53 (40, 64)	53 (42, 62)	57 (46, 69)	<0.001

*^a^*Wilcoxon rank-sum test for complex survey samples.

The results of the multivariate logistic regression models indicated a statistically significant positive correlation between the CERAD word learning and recall test scores and serum C23:0 levels as continuous measures in Models 1 (unadjusted) and 2 (adjusted for age). However, no significant association was found in Model 3 (adjusted for age, race, education level, PIR, alcohol intake, and sleep disorder). We also considered serum C23:0 levels to be categorical variables for sensitivity analysis and observed a similar trend. The analysis of serum C23:0 by quartile revealed that there is a positive association between higher serum C23:0 levels and better cognitive function (Table 3). Specifically, in Models 1 and 2, the higher serum C23:0-level group (Q4) had higher CERAD scores compared to the lower-level group (Q1), while the difference was not significant in the groups with intermediate levels (Q2 and Q3).

**TABLE 3:** Adjusted* odds ratios (95% confidence intervals) for score on CERAD word learning sub-test, for blood tricosanoic acid.

	CERAD1		CERAD2					CERAD3	CERAD.delay.recall			
	**OR**	**CI**	***p***	**OR**	**CI**	***p***	**OR**	**CI**	***p***	**OR**	**CI**	***p***
**Blood tricosanoic acid (umol/L)**												
Model1	**1.02**	**1.01, 1.03**	**0.004**	**1.03**	**1.02, 1.04**	**<0.001**	**1.03**	**1.02, 1.04**	**<0.001**	**1.04**	**1.02, 1.06**	**<0.001**
Model2	**1.02**	**1.00, 1.03**	**0.018**	**1.02**	**1.01, 1.04**	**<0.001**	**1.03**	**1.01, 1.04**	**<0.001**	**1.03**	**1.01, 1.05**	**<0.001**
Model3	1	0.98, 1.02	0.9	1.01	0.99, 1.02	0.4	1.01	1.00, 1.03	0.14	1.02	1.0, 1.04	0.13
**Quartile of blood tricosanoic acid**												
Q4 vs. Q1, model1	**1.7**	**1.25, 2.31**	**0.001**	**1.9**	**1.38, 2.62**	**<0.001**	**1.87**	**1.34, 2.59**	**<0.001**	**2.35**	**1.44, 3.82**	**0.001**
Q4 vs. Q1, model2	**1.5**	**1.13, 2.00**	**0.007**	**1.68**	**1.20, 2.36**	**0.004**	**1.69**	**1.21, 2.35**	**0.003**	**1.97**	**1.24, 3.14**	**0.006**
Q4 vs. Q1, model3	1.08	0.75, 1.57	0.7	1.09	0.74, 1.61	0.7	1.28	0.83, 1.98	0.2	1.41	0.79, 2.50	0.2
Q3 vs. Q1, model1	1.26	0.90, 1.76	0.2	1.31	0.91, 1.90	0.15	**1.44**	**1.00, 2.06**	**0.047**	**1.68**	**1.08, 2.63**	**0.023**
Q3 vs. Q1, model2	1.17	0.89, 1.55	0.2	1.22	0.89, 1.68	0.2	1.36	0.98, 1.88	0.061	**1.52**	**1.01, 2.28**	**0.043**
Q3 vs. Q1, model3	0.96	0.69, 1.32	0.8	0.87	0.66, 1.15	0.3	1.07	0.74, 1.56	0.7	1.18	0.79, 1.77	0.4
Q2 vs. Q1, model1	**1.58**	**1.10, 2.26**	**0.015**	1.41	0.97, 2.05	0.067	1.31	0.85, 2.03	0.2	1.39	0.76, 2.56	0.3
Q2 vs. Q1, model2	**1.46**	**1.08, 1.96**	**0.016**	1.31	0.93, 1.84	0.12	1.23	0.83, 1.82	0.3	1.24	0.73, 2.12	0.4
Q2 vs. Q1, model3	1.3	0.94, 1.81	0.11	1.09	0.79, 1.50	0.6	1.13	0.74, 1.72	0.6	1.12	0.65, 1.93	0.7

Bold values indicate statistically significant differences. Model 1 = Blood tricosanoic acid. Model 2 = Model 1 + Age group. Model 3 = Model 2 + Age group + Race + Educational_level + PIR.group + Alq.group + sleep.disorder.

Table 4 presents the results of the linear regression analysis examining the correlation between serum C23:0 levels and the total CERAD word learning, animal fluency, and DSST scores. The findings indicated that a higher serum C23:0 level on the continuous scale was significantly associated with elevated scores on each of the three tests. Overall, on the categorical scale, a positive correlation was observed between serum C23:0 levels and total CERAD word learning score, which was particularly obvious when comparing Q4 with Q1 in Model 1 (β = 1.795, SE = 0.385, *P* < 0.001) and Model 2 (β = 1.454, SE = 0.384, *P* < 0.001). The DSST is a valid and sensitive measure of cognitive dysfunction and can monitor the effectiveness of treatments for cognitive impairment (Jaeger, 2018). The Q4 group with the highest serum C23:0 levels showed a positive association between serum C23:0 levels and DSST compared to the Q1 group with the lowest levels in the initial unadjusted Model 1 (β = 9.607, SE = 1.754, *P* < 0.001). This association remained significant after we adjusted for age in Model 2 (β = 8.007, SE = 1.554, *P* < 0.001) and persisted even in Model 3 (β = 3.610, SE = 1.248, *P* = 0.010). However, no significant pattern of associations was observed for the animal fluency test score.

**TABLE 4:** Adjusted* beta coefficients [standard error (SE), *P*-value] for score on CERAD word learning sub-test, animal fluency test, and DSST, for blood tricosanoic acid.

	CERAD total			Animal fluency			DSST		
	β	**Std error**	***p***	β	**Std error**	***p***	β	**Std error**	***p***
**Blood tricosanoic acid (μmol/L)**									
Model1	**0.077**	**0.016**	**<0.001**	**0.058**	**0.027**	**0.042**	**0.440**	**0.070**	**<0.001**
Model2	**0.065**	**0.016**	**<0.001**	0.044	0.023	0.065	**0.380**	**0.070**	**<0.001**
Model3	0.021	0.019	0.301	0.039	0.023	0.102	**0.200**	**0.070**	**0.007**
**Quartile of blood tricosanoic acid**									
Q4 vs. Q1, model1	1.795	**0.385**	**<0.001**	**1.170**	**0.625**	**0.071**	**9.607**	**1.754**	**<0.001**
Q4 vs. Q1, model2	**1.454**	**0.384**	**<0.001**	**0.777**	**0.534**	**0.158**	**8.007**	**1.554**	**<0.001**
Q4 vs. Q1, model3	0.413	0.461	0.383	**0.839**	**0.543**	**0.141**	**3.610**	**1.248**	**0.010**
Q3 vs. Q1, model1	0.868	0.462	0.070	**1.099**	**0.655**	**0.104**	**4.744**	**1.751**	**0.011**
Q3 vs. Q1, model2	0.670	0.376	0.086	**0.870**	**0.545**	**0.122**	**3.815**	**1.530**	**0.019**
Q3 vs. Q1, model3	−0.111	0.364	0.765	1.103	0.480	0.035	0.663	1.124	0.563
Q2 vs. Q1, model1	**1.071**	**0.491**	0.038	−0.174	0.696	0.804	3.523	2.393	0.152
Q2 vs. Q1, model2	**0.848**	**0.410**	**0.048**	−0.433	0.629	0.497	2.469	2.163	0.264
Q2 vs. Q1, model3	0.462	0.382	0.243	−0.487	0.626	0.447	0.639	1.769	0.722

Bold values indicate statistically significant differences. Model 1 = blood tricosanoic acid. Model 2 = Model 1 + Age.group. Model 3 = Model 2 + Age.group + Race + Educational_level + PIR.group + Alq.group + sleep.disorder.

## 4 Discussion

The study clearly demonstrated that the expression of C23:0 decreased in the frontal cortex of AD patients, and population-based validation confirmed a strong association between C23:0 and cognitive function. Through comprehensive GEO microarray analyses and in combination with the METAFlux tool, we identified a set of metabolites associated with AD; we then focused on the relationship between C23:0 and cognitive performance. To further clarify the relationship between C23:0 and cognitive function, we utilized NHANES data. We consistently found that serum C23:0 levels were positively correlated with cognitive function. Interestingly, in patients with mild cognitive impairment, C23:0 levels were elevated relative to those in the Alzheimer’s cohort (Dhillon et al., 2023). This suggests that changes in C23:0 metabolism may occur dynamically across the progression of cognitive decline. C23:0 is a long-chain saturated fatty acid, the precise mechanisms by which it influences cognitive function remain unclear. Several potential biological mechanisms have been proposed to explain how alterations in C23:0 levels could contribute to cognitive dysfunction. First, as a component of neuronal membrane phospholipids, changes in C23:0 composition could impact the fluidity and function of neuronal membranes, thereby affecting neurotransmitter signaling and neuronal activity (Herr et al., 1996). Second, C23:0 may induce neuroinflammatory responses, leading to oxidative stress and neuronal injury, which impair cognitive processes (Gupta et al., 2012; Zhang et al., 2016). Third, C23:0 may interfere with central nervous system myelination, potentially disrupting the integrity of neural communication. For example, fatty acid binding protein 8 (FABP8) has a unique function in the organization and stabilization of the multilayered myelin sheath, as it can cooperatively stack phospholipid membranes with myelin basic protein. It can influence the formation of axonal myelin sheaths by participating in the uptake and lipid metabolism of fatty acids (Greenfield et al., 1973). Additionally, perturbations in C23:0 metabolism may disrupt neuronal energy homeostasis, compromising the viability and function of neurons. Long-chain saturated fatty acids such as C23:0, which are endogenous agonists of free fatty acid receptor 1 (FFAR1), have a high affinity for FFAR1. FFAR1 is related to neurogenesis and is associated with cognitive functions such as memory, spatial orientation, and learning (Boneva et al., 2011; Ma et al., 2008; Yamashima, 2008; Zamarbide et al., 2014). Thus, through FFAR1, C23:0 may influence cognitive function (Hara et al., 2013).

While our study provides valuable insights into the association between C23:0 and cognitive function, it is important to acknowledge several limitations. First, the cross-sectional design of the NHANES data limits our ability to infer causality. Future longitudinal studies are needed to establish a temporal relationship between C23:0 levels and cognitive changes. Second, we did not adjust for potential confounders such as genetic predispositions and detailed dietary patterns. These factors could influence the observed associations and should be considered in future research. Third, as our study relies on U.S.-based NHANES data, the generalizability of our results may be limited. Replication studies in diverse populations are needed to confirm the broader applicability of our conclusions.

## 5 Conclusion

This study provides strong evidence for an association between altered C23:0 metabolism and cognitive dysfunction, with potential implications for the underlying pathophysiology and management of cognitive disorders. Our findings suggest that maintaining optimal levels of C23:0 may be beneficial for cognitive health in older adults. Dietary interventions, such as the consumption of foods rich in 23-carbon saturated fatty acids like tuna, yellowfin tuna, and sardines, could be a potential strategy to support cognitive function. However, these dietary recommendations should be considered exploratory rather than definitive, given the current state of evidence. Further research is warranted to elucidate the precise mechanisms by which C23:0 may influence cognitive processes and to assess its clinical utility in the context of neurodegeneration.

## Data Availability

The original contributions presented in the study are included in the article/Supplementary material, further inquiries can be directed to the corresponding author/s.

## Footnotes

1
https://github.com/KChen-lab/METAFlux
